# Meta-Analysis of the Effects of Overexpressed bZIP Transcription Factors in Plants under Drought Stress

**DOI:** 10.3390/plants13030337

**Published:** 2024-01-23

**Authors:** Ran Tao, Yaqiu Liu, Su Chen, Sergey Shityakov

**Affiliations:** 1 College of Computer and Control Engineering, Northeast Forestry University, Harbin 150040, China; taoran@nefu.edu.cn; 2 State Key Laboratory of Tree Genetics and Breeding, Northeast Forestry University, Harbin 150040, China; chensu@nefu.edu.cn; 3 Laboratory of Chemoinformatics, Infochemistry Scientific Center, ITMO University, Saint-Petersburg 191002, Russia; shityakoff@hotmail.com

**Keywords:** meta-analysis, drought stress, bZIP transcription factors, overexpressed

## Abstract

The bZIP (basic leucine zipper) transcription factors have been identified as key regulators of plant responses to drought stress, which limits plant growth and yield. Overexpression of bZIP genes has shown potential in enhancing drought tolerance in various plant species. However, the constrained types of individual studies and inconsistencies among experimental approaches has resulted in a lack of statistical significance and limited the extrapolation of bZIP transcription factor overexpression for plant improvement. We conducted a meta-analysis to evaluate ten measured parameters of drought tolerance in bZIP transcription factor-expressing plants as well as moderators affecting the performance of transgenic plants. The results showed that seven parameters, including survival rate as well as the content of regulatory substances (proline accumulation, H_2_O_2_ concentration, CAT activity, POD activity, SOD activity and MDA accumulation), were most affected while the impact on physiological status indicators is not significant. In addition, donor/recipient species, treatment medium, duration and methods of simulating drought stress all significantly impacted the degree of drought stress tolerance in plants to some extent among the considered moderators. The findings underscore the potential of bZIP transcription factors as key targets for genetic engineering approaches aimed at improving plant resilience to water scarcity.

## 1. Introduction

Drought is one of the most prevalent and severe environmental stresses. It affects the absorption and transportation mechanisms of plants, and the lack of water and nutrients inhibits the formation of chlorophyll, which leads to reduced photosynthetic capacity [1]. Although plants themselves can adapt to these changes through natural selection, as the frequency and intensity of drought events have increased with global climate change, the process is still so slow as to pose a major threat to ecosystem sustainability. Therefore, understanding the underlying mechanisms of plant responses to drought stress is essential for developing strategies to improve plant resilience and productivity. Studies have shown that overexpression of certain transcription factors, such as the NAC (NAM, ATAF1/2 and CUC2) and MYB (myeloblastosis) families, significantly enhances drought tolerance in plants [2]. These transcription factors regulate the expression of stress-responsive genes involved in various physiological processes, including stomatal closure, osmotic regulation and antioxidant defence, thereby increasing the water-use efficiency of the plant and minimizing drought-induced damages.

BZIP transcription factors are a class of transcription factors with basic and leucine zip regions that bind to DNA and regulate gene transcription and are one of the largest families of transcriptional regulators in plants [3]. Numerous studies have shown that the bZIP transcription factor family is involved in multiple key pathways in plant response to drought stress. By regulating gene expression, interacting with other transcription factors and regulating phytohormone signalling, they help plants adapt to a drought environment and enhance their drought tolerance. For example, Huang et al., 2010, reported that the increased expression of the PtrABF gene, a bZIP transcription factor derived from Poncirus Trifoliata, improves tobacco’s resistance to dehydration and drought by managing reactive oxygen species (ROS) and regulating the expression of genes responsive to stress [4]. BZIP is involved in the regulation of plant response to biological stresses such as insect pests and pathogen infection through salicylic acid, jasmonic acid and ABA signal transduction pathways [5].

Meta-analysis involves statistically combining extensive datasets from numerous studies [6]. It is a systematic statistical approach that provides more statistically significant conclusions and increases understanding of the research question. Meta-analysis is widely used in various disciplinary fields, including a growing application in the field of assessing plant response to abiotic stress. A meta-analysis of transcriptome responses to salt stress at seedling stage in different rice genotypes was investigated by Kong et al., 2009, and many unreported salt-responsive genes were identified [7]. Therefore, we aimed to assess the effect of overexpression of the bZIP transcription factor in response to drought stress in plants by meta-analysis. Nine physiological and growth parameters which were measured in a large number of studies were comprehensively analysed to determine the following questions: (1) the overall effects of overexpressing bZIP genes in transgenic plants on the response under arid environments across studies; (2) the differences in the role of bZIP overexpression between drought-stressed and non-stressed plants; and (3) the conditions in the experiments that affected the impact of bZIP overexpression. The present meta-analysis provides a theoretical basis and research ideas for further exploration of the drought stress-related functions of bZIP transcription factors. It also provides a theoretical basis for crop genetic improvement and green production in agriculture. This meta-analysis is used to explore the related functions affected by bZIP transcription factors under drought stress to providing a more comprehensive understanding for future research in this field.

## 2. Results

### 2.1. Summary of Overall Effects

**Figure 1:**
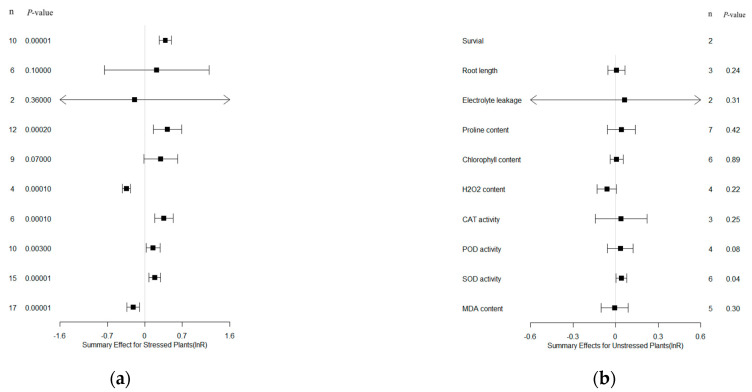
Weighted summary effect sizes (lnR) and 95% CI of the parameters for bZIP overexpression in transgenic plants subjected to drought stress (**a**) and non-stressed conditions (**b**). *p* ≤ 0.05 indicates a significant difference in the moderator level compared to zero; n represents the number corresponding to each summary effect number (same for Figure 2, Figure 3, Figure 4, Figure 5, Figure 6 and Figure 7). The arrow indicates that the upper and lower limits of 95% CI of the parameter exceeds the display range of the graph (same for Figure 2, Figure 3, Figure 4, Figure 5, Figure 6 and Figure 7).

A total of 10 summary effect sizes of bZIP overexpression in transgenic and wild-type (WT) plants, which had biological importance, subjected to drought stress and non-stress control conditions are shown in the forest plot (Figure 1). Ten different species served as gene donors, with wheat being the major donor source. Dicotyledonous plants accounted for more gene donors than monocotyledonous ones. On the other hand, there were seven types of recipient plants, all of which were dicotyledons, with *Arabidopsis thaliana* being the most studied recipient. The most commonly used promoter in all the experiments included in the analysis was CaMV 35S.

When plants were subjected to drought stress, a total of seven of the ten measured parameters were significantly affected (Figure 1a: *p* ≤ 0.05, with 95% CIs that did not intersect with the no-effect line). Under non-stressed conditions, one parameter was significant at a 95% confidence interval (SOD activity; Figure 1b). The survival rate of transformed (TC) plants was increased 130% by bZIP overexpression compared with non-transformed (NC) plants.

### 2.2. Heterogeneity Analysis

A random effects model was used to study the heterogenicity among modeler classes. Probability of the Q-test < 0.1 or I^2^ > 50% indicates a high heterogeneity between the results of studies, which means that the results of the different studies are too varied to demonstrate that the true effect is consistent with the pooled effect. This may be due to differences in participants, methodology, sample sizes and other factors across studies. Moderators or subgroup analyses are often used to further investigate the sources of heterogeneity to understand potential factors and variables. A p_prob_ value exceeding 0.1 is inadequate to establish that genuine effects align with summary effects, suggesting a lack of statistical significance [6].

Table 1 and Table 2 provide heterogeneity statistics for the summary effect sizes of nine parameters across diverse ecological conditions. Under drought stress conditions, the *p*-value for heterogeneity was significant (*p* < 0.1) for two parameters (Table 1), namely H_2_O_2_ content (*p* = 0.04967, I^2^ = 52.42%) and CAT activity (*p* = 0.0495, I^2^ = 54.94%). In total, three summary effect sizes had an I^2^ value of 0%, and five had a small positive value. Two had significant heterogeneity (*p* < 0.1), which will be subjected to further subgroup analysis. Under non-stress conditions, I^2^ < 40% for two summary effects, while all others were 0, and none of them showed significant heterogeneity. A lack of significance may occur due to a small sample size or relatively consistent measurement methods. Therefore, even if there is little statistical heterogeneity, subgroup analyses of these variables need to be conducted in conjunction with study design, data and statistical methods.

**Figure 2:**
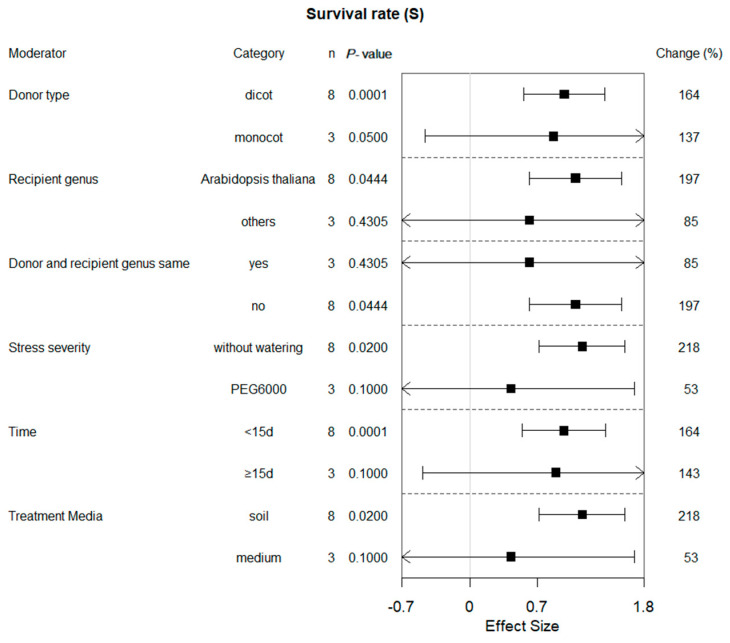
Subgroup analysis of the effects on survival rate of plants under drought-stressed conditions. Survival rate is evaluated with respect to six moderators, with each moderator level representing a distinct category.

**Figure 3:**
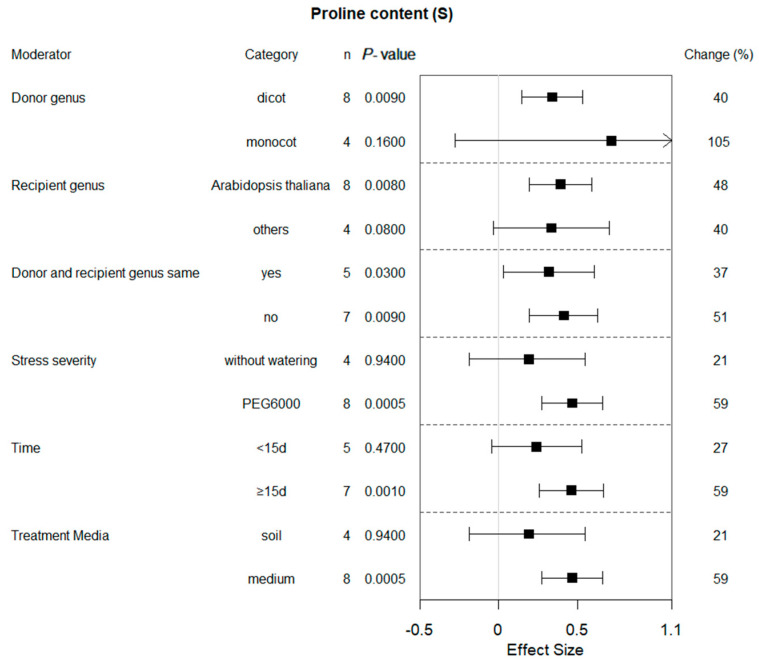
Subgroup analysis of the effects on proline content of plants under drought-stressed conditions. Six moderators influenced plant proline content under drought, with each category representing a specific moderator level.

**Figure 4:**
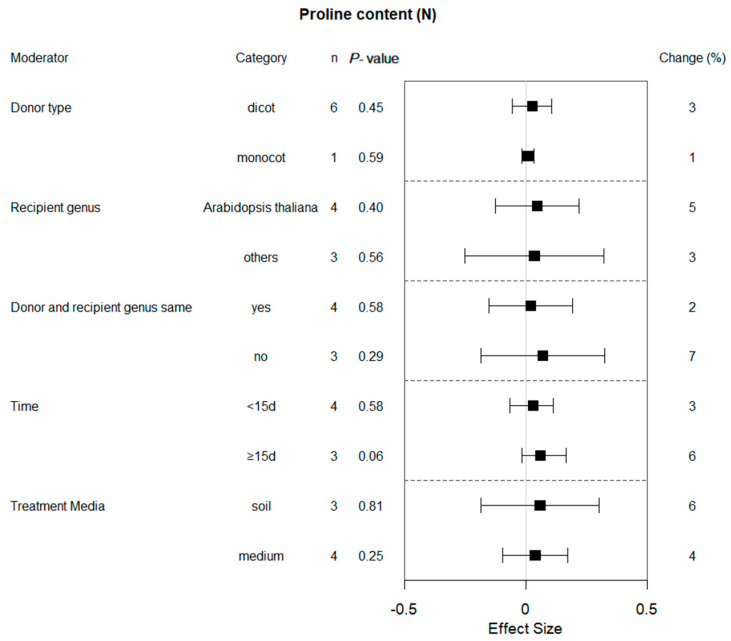
Subgroup analysis of the effects on proline content of plants under non-stressed conditions. Five moderators influenced proline content in drought-exposed plants, with each moderator level representing a distinct category.

**Figure 5:**
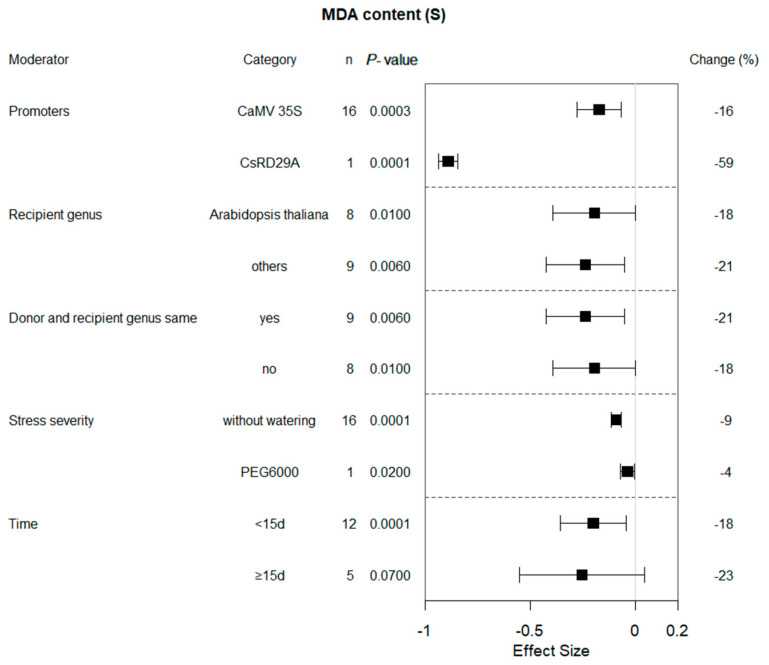
Subgroup analysis of the effects on MDA content of plants under drought-stressed conditions. Five moderators influenced MDA content in drought-exposed plants, with each moderator level representing a distinct category.

**Figure 6:**
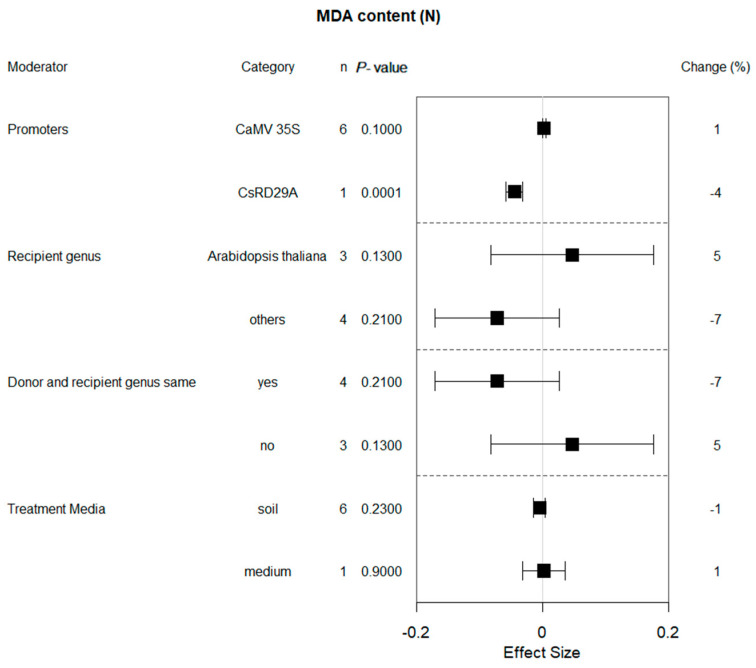
Subgroup analysis of the effects on MDA content of plants under non-stressed conditions. MDA content is evaluated with respect to four moderators, with each moderator level representing a distinct category.

**Figure 7:**
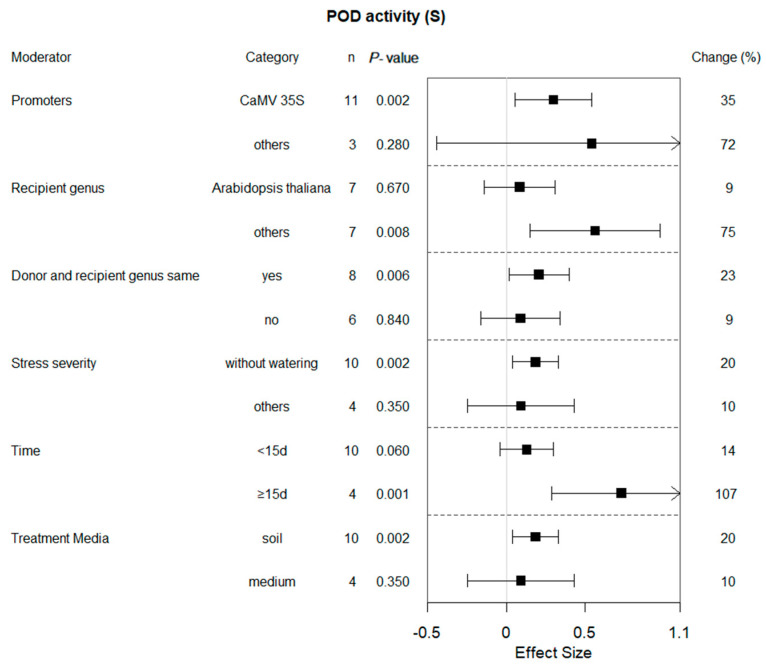
Subgroup analysis of the effects on POD activity of plants under drought-stressed conditions. POD activity is evaluated with respect to six moderators, with each moderator level representing a distinct category.

Six of the ten summary effects influenced by bZIP gene expression were selected for moderator analyses. Figure 2, Figure 3, Figure 4, Figure 5, Figure 6 and Figure 7 show the impact of moderator levels of the different variables on the summary effects.

### 2.3. Subgroup Effect within Each Moderator on Survival Rate in Plants under Drought Conditions

The specific effect of overexpression of bZIP genes on plant survival under drought conditions was significant when the recipient genus was *Arabidopsis thaliana* as well as when the donor and recipient were different (Figure 2). The survival rate was notably increased when drought was imposed by withholding irrigation. Different donor types also affected the results; for example, the rate of dicotyledonous plant donors has been more significantly influenced. A higher influence on survival was observed in soil-grown transgenic plants than that in medium. If bZIP overexpressing plants were exposed to drought stress for <15 d, there was a significant positive moderating effect on the survival of transgenic plants. There were also two studies in which all of the WT plants died under drought stress, and, therefore, they were not counted in the statistics.

### 2.4. Subgroup Effect within Each Moderator on Proline Content in Plants That Experienced Drought and Non-Stressed Conditions

Monocotyledonous plants act as the donor genus, and water deficiency did not affect the proline content under drought conditions at 95% confidence intervals (Figure 3). When plants were cultivated and assessed with PEG6000, the effect of transformation was approximately threefold of that without watering. In contrast to plants grown in soil, transformation had a much larger impact on proline content when the transgenic plants were grown in medium. When the donor and recipient genus were same and the recipient species was *Arabidopsis*, the proline content of transgenic plants exhibited higher levels compared to the WT plants. The proline content of transgenic plants was significantly increased by 27% and 59% when exposed to drought stress for <15 d and ≥15 d, respectively. However, no difference was found in these moderators under non-stress conditions, and the transgenic plants had almost similar effects on proline levels (Figure 4).

### 2.5. Moderator Analysis of MDA Content in Plants Subjected to Drought and Non-Stressed Conditions

The MDA activity of transgenic and WT plants under drought and non-stress conditions were different (*p* = 0.00001 and *p* = 0.3, respectively; Figure 1). When donors and receptors came from different genera and *Arabidopsis* served as the recipient genus, transformation reduced the MDA content by 18% (Figure 5). MDA activity was lower when stopping watering the plants compared to PEG treatment. The duration of drought treatment also affected the influence of bZIP transcription factors on MDA content. A significant impact of transformation on the reduction of MDA activity was observed when exposure times of less than 15 days were used. The bZIP gene expression driven by the CaMV 35S promoter had different effects on transgenic plants under non-stress versus drought stress conditions (Figure 6). In addition, proline content in non-stress transgenic plants was almost unaffected by growth conditions.

### 2.6. Subgroup Analysis of POD Activity in Plants under Drought Conditions

When the bZIP gene was expressed driven by the CaMV 35S promoter, the POD level was higher than that driven by other promoters (Figure 7). POD levels increased about eight-fold in transgenic plants that stopped watering compared to other osmotic stress treatments (PEG6000, mannitol). If both the gene donor and recipient species belonged to the same genera, it may be considered that overexpressing the bZIP transcription factors might increase POD activity. For lengths of stress duration, those plants exposed for greater than or equal to 15 d exhibited significantly higher POD activity compared to those exposed for less than 15 days. The protective effect produced by bZip overexpression was enhanced as time increased. In addition, soil as the treatment medium facilitated POD activity with a promoting effect of 89%.

### 2.7. Subgroup Analysis of CAT Activity in Plants under Drought Conditions

**Figure 8:**
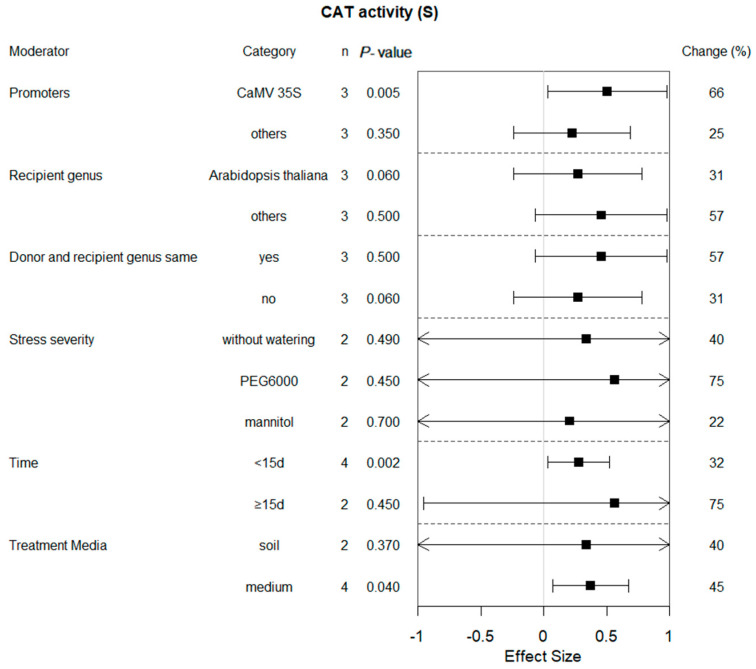
Subgroup analysis of the effects on CAT activity of plants under drought-stressed conditions. Six moderators influenced CAT activity in drought-exposed plants, with each moderator level representing a distinct category.

The impact of drought on CAT activity is not significantly related to the classification of recipient genera or whether they are consistent with the donors (Figure 8). Regarding the promoter, compared with other promoters, it achieved more than twice the induction when CaMV 35S was used as a promoter. The same trend was observed when the treatment time was less than 15 days. As a whole, different treatments had a comparable level of influence on drought response.

### 2.8. Subgroup Analysis of H_2_O_2_ Content in Plants under Drought Conditions

**Figure 9:**
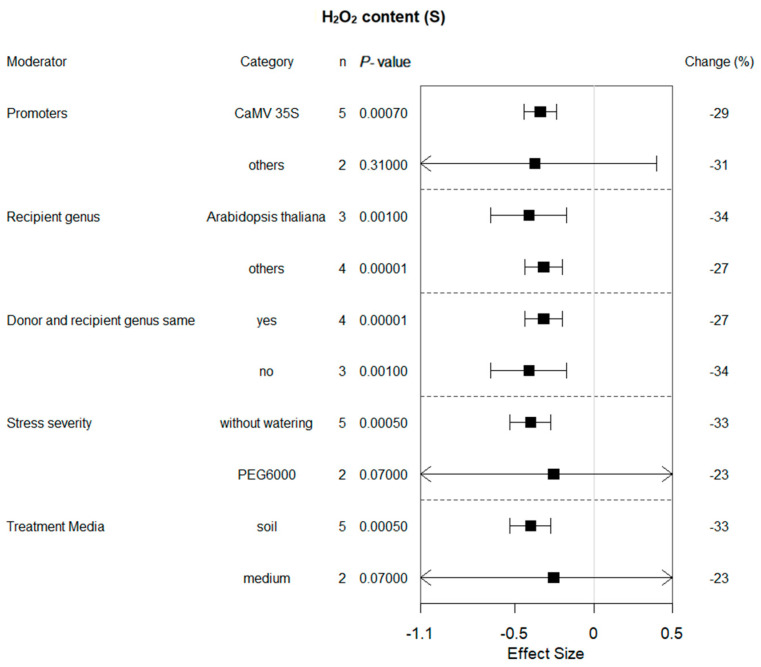
Subgroup analysis of the effects on H_2_O_2_ content of plants under drought-stressed conditions. CAT activity is evaluated with respect to five moderators, with each moderator level representing a distinct category.

H_2_O_2_ content showed only slight changes with most moderators (Figure 9). The effects of bZIP on H_2_O_2_ content under drought were sensitive to whether donor and recipient genus were the same, as well as the taxonomic traits of receiver plants. It is transparent to see that a more significant impact was obtained when stress was imposed by withholding irrigation and the treatment media was soil compared to PEG treatments and medium media.

## 3. Discussion

Multiple research results can be comprehensively evaluated to obtain a more accurate estimate of the overall effect of bZIP overexpression by meta-analysis on drought stress, including the extent of both positive and negative effects. Additionally, meta-analysis can identify possible study bias, heterogeneity and differences in specific conditions, helping to determine which environmental factors have the greatest regulatory effect on the effects of bZIP transcription factor overexpression. It is well-known that bZIP transcription factors perceive and respond to drought signals and regulate the expression of several drought-related genes. In this process, bZIP transcription factors interact with other proteins such as kinases, other transcription factors and molecules associated with drought signaling [8]. Our meta-analysis revealed a significant positive effect of overexpressing bZIP transcription factors on plant drought stress response. Parameters with strong operability and significant biological significance were selected for analysis. The analysis of the pooled data indicated that plants with bZIP overexpression exhibited a higher survival rate, increased proline content, weakened H_2_O_2_ content and reduced MDA activity under drought conditions compared to control plants. Huang et. al.’s 2010 study on overexpression of the *PtrABF* gene, *Poncirus trifoliata*, showed that it functions in positive modulation of drought stress tolerance in tobacco via scavenging ROS and modulating expression of stress-responsive genes [4]. Yan Yang et al., 2020, overexpressed GmbZIP2 in soybeans and found that the transgenic plants exhibited a survival rate four times greater than that of WT plants following a two-week drought treatment [9]. Shiqin Yang et al., 2019, show that the WT plant had a significantly lower recovery than the *OsbZIP62V* plants treated with 20% PEG for 3 days did, after which they recovered for 4 days [10]. Our meta-analysis showed that transgenic plants had a superior ability to survive drought compared to wild-type plants (*p* < 0.00001). The majority of researchers in individual studies reached the consensus that their transgenic plants exhibited greater rates of survival compared to the corresponding wild-type (WT) plants [11,12,13,14,15,16]. The results of many independent studies were combined, and moderator analysis was conducted. We found that all experimental promoters included in the survival study were the *Cauliflower mosaic virus* CaMV35S promoter which was probably due to the strong transcriptional activity of *Cauliflower mosaic virus* [17]. Therefore, many researchers have chosen to use it to achieve high expression of target genes. Moreover, most researchers preferred *Arabidopsis thaliana* as a recipient genus or PEG6000 as stress severity as well as soil as treatment media when evaluating the survival of transgenic plants expressing the bZIP gene under drought conditions, which resulted in the limited sample size of the other moderator. Therefore, it is necessary to further collect more samples for research to obtain sufficient statistical capabilities.

Malondialdehyde is a product of lipid peroxidation reaction. Measurement of MDA content is an indicator to evaluate the extent of oxidative damage to plant cell membranes under drought conditions. Drought-resistant plants can cope with drought-induced oxidative stress and cell membrane damage better, thereby reducing lipid peroxidation in the cell membranes and decreasing the accumulation of MDA accumulation [18]. Yafei Li et al., 2022, studied *CsbZIP50*-overexpressing plants and found *CsbZIP50* exhibited enhanced drought tolerance compared with wild-type plants, as evidenced by higher antioxidant enzyme activities and lower levels of reactive oxygen species (ROS) and MDA content [19]. Other researchers have had similar results [9,11,20,21,22,23]. Based on various individual studies, meta-analysis showed that overexpression of bZIP genes reduced the MDA content level of plants under drought stress. Under normal growth conditions, there was no difference in MDA content between transgenic and wild-type plants. The effect of bZIP overexpression was also influenced by several different regulatory factors (Figure 5). It is worth mentioning that the original percentage of MDA content actually increases when PEG is used to simulate drought stress conditions. Plant roots absorb impurities, such as sodium and phosphorus from PEG, which can affect plant reactions [24]. Hence, holistic consideration should be given to compounds used to simulate drought stress when studying the potential effects of drought stress tolerance levels in genetically modified plants.

Plants regulate intracellular osmotic pressure through cellular synthesis and accumulation of proline to reduce the inhibitory effects of environmental stress on plant growth [25]. In water-stressed plants, the concentration of proline in plants increased significantly [26]. Chen Kang et al., 2019, overexpressed IbbZIP1 in sweet potato, and the transgenic *Arabidopsis* plants showed an increase in ABA and proline contents and superoxide dismutase activity and a significant decrease of H_2_O_2_ content under drought stresses [27]. Overexpression of *FtbZIP83* in Tartary buckwheat had significantly higher content of proline in transgenic lines than that in WT plants [11]. Similar conclusions have been reached in other species by other researchers, such as wheat [12,13], tomato [28] and *Populus trichocarpa* [21]. These findings are also consistent with our meta-analysis results. Meanwhile, the effect of bZIP overexpression on proline accumulation may also be moderated by different conditions, particularly by the taxonomic features of the donor. Our meta-analysis shows that bZIP genes obtained from monocots perform much better than those obtained from dicots. Similar results have been reported in several studies. The addition of exogenous proline made the tolerance of a monocot to water stress more improved compared to a dicot [29]. However, our meta-analysis also illustrated significant changes in proline levels even in the non-stress conditions. Most individual studies are also consistent, with similar results to ours [11,19,21]. This indicates that bZIP transcription factors may directly regulate genes involved in osmotic regulation and antioxidant responses, such as proline biosynthetic pyrroline-5-carboxylate synthase (P5CS). Therefore, overexpression of bZIP genes induced proline accumulation and a physiological response similar to that of drought stress observed even under non-stress conditions. The physiological status of a plant elicits a response in the form of proline synthesis and accumulation [30]. Concurrently, some researchers hold the opposite view, suggesting that some bZIP overexpressing transgenic plants have lower proline levels under drought conditions compared to those in wild-type plants. Yanglu Pan et al., 2017, considered *SlbZIP38* to be a negative regulator of drought resistance because *SlbZIP38* overexpression reduced the chlorophyll and free proline content in leaves but increased the malondialdehyde content, which may explain the reduced drought tolerance observed in these lines [31]. It illustrates that the bZIP protein is a family of transcription factors that regulate multiple responses to abiotic stress. BZIP overexpression may induce a variety of results regarding upregulation or reduction in proline content; thus, additional research is still needed to enhance statistical capabilities for the analysis and supplementation of this variable.

In general, the effect of POD activities in transgenic and WT plants in arid environments was positive, and it was similar under non-stress conditions. This indicates that transgenic plants have stronger stress resistance. SOD and POD activities increased in overexpressed lines in PEG-induced stress than in the WT of *Populus* [21]. Many researchers have also reached similar conclusions [11,19,20,32]. However, Man Zhang et al., 2020, found that there was a marked decrease in POD and CAT activities in the *OX-GmbZIP15* soybean plant compared to WT plants and that *GmbZIP15* functions as a negative regulator in response to salt and drought stresses [33].The variations could be attributed to variances in the research subjects and materials employed. Consequently, more studies are required to support this conclusion. The meta-analysis clearly demonstrated that various factors, such as the type of promoter employed, recipient genus and duration of time, significantly impacted POD activity levels of the transgenes in the recipient plants.

Excessive accumulation of reactive oxygen species (O_2_^−^ and H_2_O_2_) in plants can result in cellular oxidative damage, such as leakage of cell membranes when oxidative stress occurs [34]. In addition to the aforementioned phenotypic indicators, there are other measured parameters that exhibit sensitivity towards arid environments. Among them, the content of H_2_O_2_ in transgenic plants was significantly reduced as an overall effect, while CAT activity, which is one of the enzymes mainly responsible for clearing hydrogen peroxide (H_2_O_2_) in plants, has the opposite regulatory effect. For instance, overexpression of *TaFDL2-1A* in transgenic wheat and the introduction of the maize *GhABF2* gene into cotton leads to higher antioxidant enzyme activities under drought conditions, conferring greater drought tolerance [22,35]. Overexpression of *GhABF3* in cotton significantly increased the CAT activity of the transgenic plants under drought growth conditions [36]. Lower MDA and H_2_O_2_ contents of transgenic *Arabidopsis* plants than WT plants indicate that transgenic plants have greater tolerance to dehydration stress [27,37].

In the integration analysis of measuring proline content, survival rate, H_2_O_2_ content and MDA activity, our meta-analysis results revealed that *Arabidopsis* exerted the most prominent impact as the recipient genus. Nevertheless, *Arabidopsis thaliana* was used as the recipient genus in all measurement studies when the donor and recipient genus were different. *Arabidopsis*, as a model plant and sensitive to stress, has a higher level of research than other plants. However, this may also limit the broad applicability of the studies for meta-analysis. More in-depth subgroup analyses will be considered in subsequent analyses to gain a deeper insight and explain the importance of bZIP family genes in plants.

Moreover, SOD activity also had a significant positive moderating effect, and there was no significant heterogeneity among the studies. However, a lack of sufficient reports prevented a detailed analysis of moderators. In addition, other parameters without a significant difference between transgenic and WT plants were not further examined (Figure 1). Therefore, it is still necessary to explore other statistical methods to comprehensively explain and understand how overexpression of bZIP transcription factors affect them under drought conditions. Furthermore, although we selected these parameters, they may be related to multiple stress conditions. This can provide interesting directions for future research to further explore the role of these parameters under different stress conditions.

## 4. Materials and Methods

### 4.1. Data Collection

Endnote X9 (Thomson Scientific Company, Stanford, CT, USA) was used to conduct a literature search using three electronic databases—Scopus, PubMed and Web of Science on 18 October 2023. The keywords (“bZIP transcription factors”) and (“drought” or “abiotic stress”) and (“overexpression” or “over-express”) and (“woods” or “plant”) were used. The starting list contained 310 papers, of which 39 were eliminated because of duplication. A total of 251 did not fully meet the following criteria:

- It should be written in English;
- It should be published between 2013 and 2023;
- It should quantitatively assess the effect of drought on at least some outcome parameters of interest (such as proline content and survival rate);
- Mutant plants or co-expressed with other genes are not considered;
- It is not a review or meeting report.

**Figure 10:**
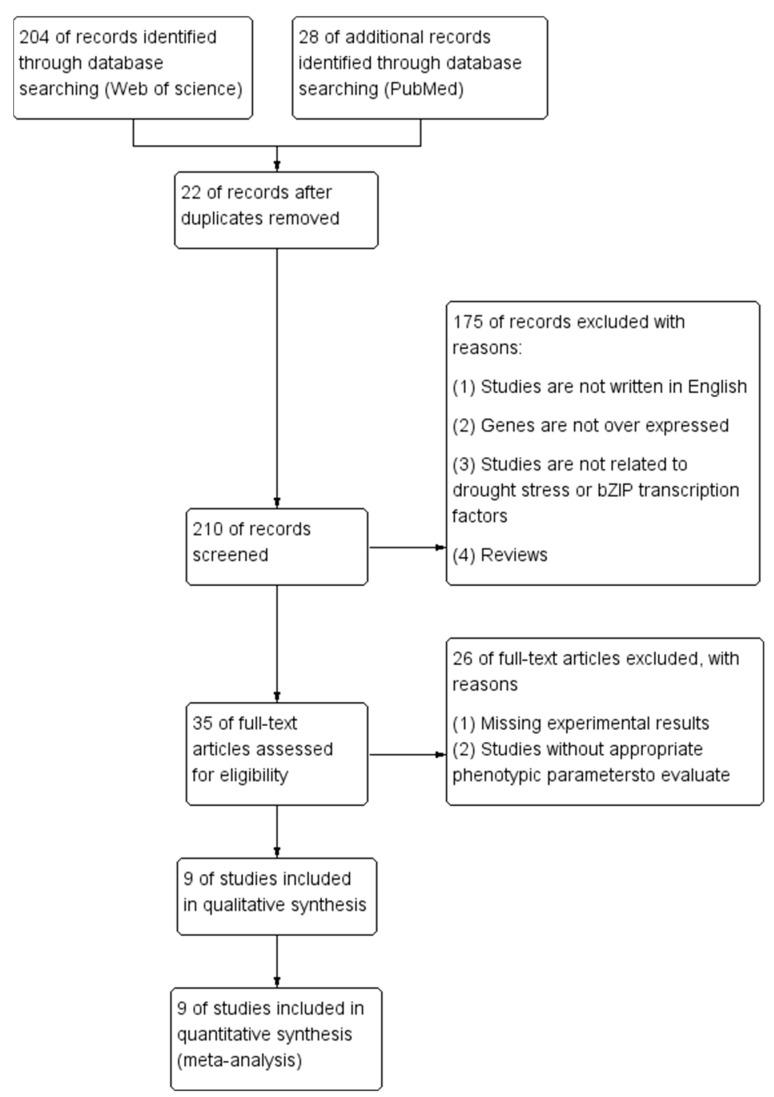
Study selection flow chart.

In total, eight studies were removed as they were non-research articles (7 reviews and one conference abstract); Seven studies were related to mutant plants; data unrelated to drought stress, 38; Forty-four were not related to bZIP transcription factors; Eight were written in other languages; Ninety studies were excluded because bZIP genes not overexpressed; drought stress or overexpression of bZIP transcription factors was not the priority of 56 studies. The remaining 20 studies, which are decade-spanning, were included for meta-analysis (Supplementary Table S1). A sum of 60 separate studies were derived from these papers (Figure 10). If various treatments existed in the paper, each of them was considered an independent study and treated as a distinct entity in the meta-analysis [38].

### 4.2. Effect Size and Moderator

Several meta-analyses were conducted on parameters measured in published studies focused on drought adaptation. We extracted the mean, sample size (n) and standard deviation (SD) of the response to exposure to drought or normal environmental conditions in transgenic plants and corresponding wild-type (WT) plants from each study. If the ‘sample size’ was not specified beforehand, it is prudent to assume that they are n = 3. For studies that included multiple time points, measurements were collected for each time point. The “juicr” package in R was used to determine the actual values when data were presented as pictures only. The data are expressed as the natural logarithm (lnR) of the response ratio (R) of transgenics to non-transgenics to compare plant responses to drought stress in different studies and to obtain an overall effect size by combining effect sizes:

lnR = lnY_TC_/Y_NC_, (1)

where Y_TC_ and Y_NC,_ respectively, represent the mean of plants that were transformed (TC) and non-transformed (NC) with bZIP overexpression. This is an indicator that is now widely used in plant science meta-analyses [39].The mean of plants with an empty vector was also incorporated as a control (NC). The variable lnR is usually used to obtain a standardized measurement of treatment-induced effects, which be used to evaluate the influence of a factor on the induction and whether the influence is significant [30,40,41]. The sign (positive or negative) of the overall effect quantity indicates whether the TC-induced conditions have a positive impact on the parameters. The absolute value of the overall effect size reflects the magnitude of the effect.

In addition to the size of the effect, detailed information on experimental variables that may affect plant responses to drought stress was also collected from each study to evaluate the heterogeneity between research results. The moderators were collected from two variables: (a) experimental conditions—stress type, stress time, treatment medium; (b) experimental materials—promoters, the type and genus of both donor and recipient and whether the recipient and donor type were the same genus. Each moderator was set to at least two levels. To meet the criteria for analysis, each moderator must encompass a minimum of three studies across more than one published report. In cases where moderators did not meet these requirements, they were assigned to a separate level called “other”, which should also meet the above criteria. Moderators who failed to meet the aforementioned criteria were not included in the analysis.

### 4.3. Meta-Analysis

After rigorous data selection and quality assessment, Metawin software 2.1 and R4.3.0 was used to perform statistical analyses and create forest plots. This enabled us to determine the overall effect size and evaluate the heterogeneity among the included studies. The separate studies were allocated weights through the utilization of a non-parametric variance approach:

VlnR = (n_TC_ + n_NC_)/(n_TC_ × n_NC_) (2)

where VlnR is the variance of natural log of response rate (R). n_TC_ is the number of transgenic samples, and n_NC_ represents the count of non-transgenic samples or transgenic samples with an empty vector [6]. A random effects model was conducted in all analyses. The summary effect size was considered significant if *p* < 0.05. If the 95% confidence interval (CI) on the forest plot intersects with the value of 0, indicating that the line representing the particular parameter level crosses the vertical line, then the observed difference between the treatment and non-treatment groups is deemed statistically non-significant. Conversely, if no intersection exists, there is 95% confidence that the difference between the two treatments is significant. The line segments around each block represent the confidence interval for effect estimation. This interval represents the credibility of our estimation of the effect. Longer line segments represent greater uncertainty, while shorter line segments represent higher accuracy.

The Q statistic is used to test for heterogeneity of effects between studies. It is used to determine whether the study effects are consistent across the board by calculating the differences between them [6,42]. If the *p*-value of Q is <0.1, it indicates that there is statistical heterogeneity between studies, which means that the effect varies across studies. On the other hand, I^2^ is a measure of statistical heterogeneity, which indicates the degree of variation in the results of a study due to heterogeneity. The value of I^2^ ranges from 0% to 100%. Generally, a value of I^2^ greater than 50% is considered to be associated with a high degree of heterogeneity, whereas a value of less than 25% is considered to be associated with a low probability of heterogeneity.

## 5. Conclusions

The overexpression of bZIP transcription factors represents a promising avenue for mitigating the adverse effects of drought stress on plants. Overall, our meta-analysis showed that overexpression of the bZIP transcription factor significantly enhanced plant tolerance to drought stress. Furthermore, subgroup analyses based on different plant species, types of bZIP transcription factors and experimental conditions provided valuable insights into the factors influencing the effectiveness of bZIP overexpression. However, due to the limited sample size involved in evaluating certain types of regulatory factors and the possibility that unpublished bZIP overexpression studies may alter the results of the meta-analysis, it is necessary to include more research to further explore the specific mechanisms of bZIP overexpression in plants under water stress conditions. Finally, these findings also provide a theoretical basis for further research on methods to reinforce plant stress tolerance when facing climate change.

## Figures and Tables

**Table 1:** Heterogeneity statistics for 10 summary effect sizes under drought-stressed conditions.

Trait	Qt	*p*	I^2^ (%)
Survival	18.714	0.044	46.56
Root length	4.3180	0.5046	0
Electrolyte leakage	1	0.31731	0.00
Proline content	19.7831	0.04841	44.40
Chlorophyll content	9.8793	0.2736	19.02
H_2_O_2_ content	12.6098	0.04967	52.42
CAT activity	11.0961	0.04951	54.94
POD activity	13.6856	0.39635	5.01
SOD activity	7.9343	0.89272	0
MDA content	26.4189	0.04841	39.44

Qt represents total heterogeneity; P_prob_ that Qt was due entirely to sampling error and not to variation among true effects; I^2^ percentage of heterogeneity due to variation among true effects (same for Table 2).

**Table 2:** Heterogeneity statistics for 10 summary effect sizes under non-stressed conditions.

Trait	Qt	*p*	I^2^ (%)
Survival			
Root length	3.244	0.3555	7.52
Electrolyte leakage	0.1628	0.68656	0
Proline content	4.6492	0.58952	0
Chlorophyll content	4.9435	0.42282	0
H_2_O_2_ content	3.7056	0.59253	0
CAT activity	1.3531	0.50838	0
POD activity	1.5722	0.66571	0
SOD activity	7.3340	0.19696	31.82
MDA content	3.6885	0.71874	0

## Data Availability

Data is contained within the article and Supplementary Materials.

## Supplementary Materials

The following supporting information can be downloaded at https://www.mdpi.com/article/10.3390/plants13030337/s1, Table S1: The detailed data of original bZIP overexpression studies.
